# Sarcopenia Is Independently Associated with Cardiovascular Disease in Older Korean Adults: The Korea National Health and Nutrition Examination Survey (KNHANES) from 2009

**DOI:** 10.1371/journal.pone.0060119

**Published:** 2013-03-22

**Authors:** Sang Ouk Chin, Sang Youl Rhee, Suk Chon, You-Cheol Hwang, In-Kyung Jeong, Seungjoon Oh, Kyu Jeung Ahn, Ho Yeon Chung, Jeong-taek Woo, Sung-Woon Kim, Jin-Woo Kim, Young Seol Kim, Hong-Yup Ahn

**Affiliations:** 1 Department of Endocrinology and Metabolism, Kyung Hee University School of Medicine, Seoul, Korea; 2 Department of Endocrinology and Metabolism, Kyung Hee University Hospital at Gangdong, Kyung Hee University School of Medicine, Seoul, Korea; 3 Department of Statistics, Dongguk University, Seoul, Korea; College of Pharmacy, University of Florida, United States of America

## Abstract

**Background:**

The association between sarcopenia and cardiovascular disease (CVD) in elderly people has not been adequately assessed. The aim of this study was to investigate whether CVD is more prevalent in subjects with sarcopenia independent of other well-established cardiovascular risk factors in older Korean adults.

**Method:**

This study utilized the representative Korean population data from the Korea National Health and Nutrition Examination Survey (KNHANES) which was conducted in 2009. Subjects older than 65 years of age with appendicular skeletal muscle mass (ASM) determined by dual energy X-ray absorptiometry were selected. The prevalence of sarcopenia in the older Korean adults was investigated, and it was determined whether sarcopenia is associated with CVD independent of other well-known risk factors.

**Results:**

1,578 subjects aged 65 years and older with the data for ASM were selected, and the overall prevalence of sarcopenia was 30.3% in men and 29.3% in women. Most of the risk factors for CVD such as age, waist circumference, body mass index, fasting plasma glucose and total cholesterol showed significant negative correlations with the ratio between appendicular skeletal muscle mass and body weight. Multiple logistic regression analysis demonstrated that sarcopenia was associated with CVD independent of other well-documented risk factors, renal function and medications (OR, 1.768; 95% CI, 1.075–2.909, *P* = 0.025).

**Conclusions:**

Sarcopenia was associated with the presence of CVD independent of other cardiovascular risk factors after adjusting renal function and medications.

## Introduction

Sarcopenia refers to the loss of skeletal muscle mass and strength that occurs with normal aging and may be of great importance, as it can lead to disability and chronic complications [1], [2]. Its prevalence varies between 7 to 24% [3]–[8], possibly due to the absence of an accepted definition of sarcopenia necessary for research and clinical purposes as well as heterogeneous study populations.

Sarcopenia is believed to be a part of the ageing process [9]. With fat infiltration deposited within muscle fibers [10], a loss of motor units as well as atrophy of the type II fast glycolytic fibers is known to accelerate the development of sarcopenia, and as a consequence, muscle power necessary for daily life is gradually lost and physical activity decreases [11]. In addition, chronic inflammation and catabolic cytokine production have been also reported to play significant roles in the development of sarcopenia [12]. All of these processes are highly linked to its detrimental metabolic effects causing a higher prevalence of chronic diseases such as type 2 diabetes mellitus (DM), hyperlipidemia and hypertension (HTN) [13] which are well-known risk factors for cardiovascular disease (CVD).

However, there have been few studies examining the relationship between sarcopenia and CVD, and the results are conflicting [14]–[19]. A Japanese study demonstrated that sarcopenia was significantly associated with greater arterial stiffness especially in women, which did not directly analyze between sarcopenia and CVD [14]. Also, the similar result was found when muscle mass was indirectly measured using 24 h urinary creatinine excretion rates instead of dual energy X-ray absorptiometry (DXA) [18], [19]. On the other hand, Aubertin-Leheudre et al., found that sarcopenia was associated with lower risk factors predisposing to CVD but their study populations had very unique features such as including only postmenopausal women [15]. Sarcopenia with combined obesity was reported not to be associated higher risk of functional limitation in older patients, but in this study muscle mass was estimated by using the predicted equation [16], thus making their results not easily generalizable or clinically useful.

Considering the absence of appropriate and consistent evidence regarding the association between sarcopenia and CVD, the aim of the present study was to determine whether or not sarcopenia is associated with CVD independent of other well-established CVD risk factors in Korean adults over 65 years old based upon data from the Korea National Health and Nutrition Examination Survey (KNHANES) IV, which was conducted in 2009.

## Materials and Methods

### Ethics Statement

This study was approved by the Institutional Review Board of Kyung Hee University Hospital at Gangdong (approval number KHNMC 2012–128). When KNHANES IV was conducted, all of the participants were informed that they had been randomly chosen to participate in the survey with the right to refuse to participate in the further analyses, and signed informed consents.

### KNHANES IV

KNHANES is a nationwide, population-based, cross-sectional health survey. As of its first survey in 1998, KNHANES IV was performed between July 2007 and December 2009, which used a randomized selection method choosing from all households recorded in the Population and Housing Census in Korea in 2005. The entire nation was divided into 29 ranks based upon administrative districts and housing types. Two hundred districts from each rank were randomized by applying proportional allocation to equalize the distribution ratio between the entire population and the samples within each rank. One survey section from each district was selected based upon the characteristics of housing types and 20–23 households from each survey section were chosen by a rolling sampling system to represent all parts of the nation. This method assured the homogeneous and independent characteristics of all sampled households.

The subjects of KNHANES IV included all members of the sampled households >1 year of age. The questionnaire was composed of a health examination and interview, and a nutritional interview. A total of 31,705 KNHANES IV sampled subjects in 9,421 households were screened. Among them, 23,632 (74.5%) participated in the health examination and interview.

### Definition of Sarcopenia and Obesity

This study utilized the data of KNHANES IV of subjects older than 65 years of age with appendicular skeletal muscle mass (ASM) determined by DXA (Discovery-W™; Hologic®, Inc. Bedford, MA). ASM was defined as the sum of the lean soft tissue masses of the arms and legs [20]. The precision of DXA has been previously reported. In the National Health and Nutrition Examination Survey (NHANES), DXA instruments were calibrated according the previously proposed method by Schoeller et al. [21]. The reference values of the NHANES were obtained by the calibration method previously published by Kelly et al. [22]. This calibration method was periodically applied for the appropriate comparison between the present and previous data. In addition, DXA calibrations were maintained through an internal referencing system, which regularly measured bone and soft tissue equivalent reference standards during the examination. A subject was classified as having sarcopenia when having an ASM/weight (kg) less than 1 SD below the gender-specific mean of a younger reference group between 20 and 39 years of age (1,017 men and 1,284 women), which was modified from the studies of Janssen et al. and Lim et al. [4], [23]. For men, the cutoff value for sarcopenia was 32.2% (ASM/weight), defined as less than 1 SD below the sex-specific normal mean for the young reference group. For women, the corresponding cutoff value was 25.6% (ASM/weight). A subject was classified as obese if one had a BMI greater than 25 kg/m^2^ [24]. SO was defined when a subject satisfied the criteria for both sarcopenia and obesity.

### Assessment of Cardiovascular Risk Factors

The presence of CVD events was ascertained from the health interview survey in KNHANES IV. Those who answered “yes” to the question; “Have you ever been diagnosed with either acute myocardial infarction, unstable or stable angina, or stroke by a physician or a health care professional?” were classified as subjects with previous CVD events. Subjects with DM were defined as those who were identified in the health interview survey as having a previous diagnosis of DM by a health care professional, and those satisfying the criteria provided by the American Diabetic Association as follows: glycated hemoglobin (HbA1c; measured using high-performance liquid chromatography (BIO-RAD VARIAN™ II; BIO-RAD, Hercules, CA) ≥6.5% or a fasting plasma glucose (measured by ADVIA® 1650; Siemens, Deerfield, IL) ≥126 mg/dl [25]. Insulin level was measured using a γ counter (1470 Wizard, Perkin-Elmer Finland) with an immunoradiometric assay (Biosource, Belgium). The homeostasis model assessment 2– insulin resistance (HOMA2-IR) was calculated using the equation proposed by Levy et al. [26]. Subjects with HTN included those who had either a systolic blood pressure greater than 140 mmHg or diastolic blood pressure greater than 90 mmHg. Data regarding blood pressure in KNHANES IV consisted of the mean of three separately measured blood pressures. In addition, subjects taking antihypertensive medications and those who carried a diagnosis of HTN were also included. Hyperlipidemia was defined using the criteria established by the National Cholesterol Education Program (NCEP) Adult Treatment Panel III as the following: total cholesterol (T-chol) ≥240 mg/dl or triglycerides (TG) ≥200 mg/dl [27] (all measured by ADVIA 1650™). Also, those who were taking lipid-lowering drugs or who had a previous diagnosis of hyperlipidemia were included. Metabolic syndrome (MetS) was defined according to the NCEP criteria using the Asia-Pacific abdominal obesity criteria (waist circumference ≥90 cm in men and ≥80 cm in women) [27]. Serum creatinine was measured using the same instrument for a fasting plasma glucose level.

Blood samples were collected from all subjects after >8 hours of fasting. Specimens were immediately centrifuged, aliquoted, frozen at −70°C, and moved to the central laboratory (NeoDIN Medical Institute, Seoul, Korea), where they were analyzed within 24 hours. To measure high density lipoprotein cholesterol (HDL-C), a frozen sample was prepared according to the guidelines from the Clinical and Laboratory Standard Institutes (CLSI) C37-A. These samples were analyzed in the Lipid Standardization Program (LSP) of the Centers for Disease Control and Prevention (CDC) in United States, and these values were compared with those measured by the central laboratory. Differences between these two measurements were adjusted by Passing Bablok regression. The inter-assay coefficients of variation were 0.87–3.28% for total cholesterol, and 0.86–3.95% for triglyceride.

### Statistical Analysis

All sampling and weight variables were stratified, and the statistical analyses were performed using STATA (version 10.1; StataCorp. Texas, USA). In order to calculate the total population that the sample would represent, we employed the stratification variables and sampling weights designated by the Korean Centers for Disease Control and Prevention. The sampling weights were adjusted for nonresponse according to demographic factors after surveys were completed. The total number of adults older than 65 years was calculated to be 4,888,503 people in 2009 (2,020,157 men and 2,864346 women). Statistics were used to describe the demographics and CVD risk factors of all the samples according to the sarcopenic body fat classification in either non-obese or obese groups, which were tested for statistical significance by applying the Student's t-test for continuous variables. For categorical variables, chi-squared tests were performed to evaluate the difference between each group. Spearman correlation coefficients were calculated to explore the association between CVD risk factors and ASM/weight. Linear by linear association was performed to observe the trend between the prevalence of sarcopenia in each age group by gender. Multivariate logistic regression analyses with backward selection were also used to determine whether or not sarcopenia would be independently associated with CVD. A p-value <0.05 indicated statistical significance.

## Results

### Prevalence of sarcopenia

**Figure 1:**
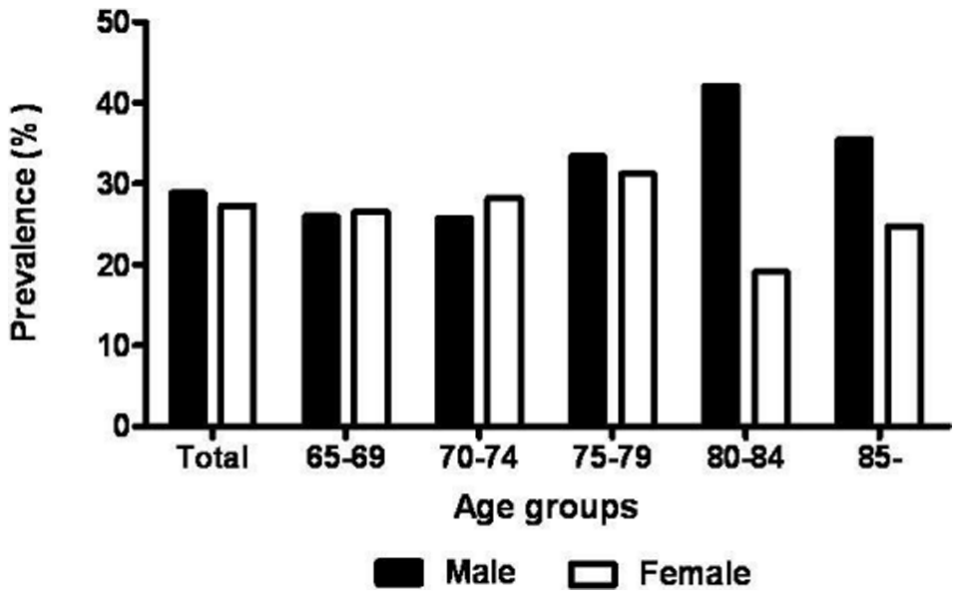
Prevalence of sarcopenia by gender and age groups; The prevalence trend according to the age group was not significant either in male (*P* = 0.092) or in female (*P* = 0.512).

Among 23,632 subjects who completed the health examination and interview, 10,533 subjects underwent DXA to measure their ASM, and 1,578 of these patients older than 65 years were included in this study. Figure 1 shows the prevalence of sarcopenia according to gender and age. The overall prevalence of sarcopenia was 30.3% and 29.3% in men and women older than 65 years, respectively. The prevalence trend according to the age group was not significant either in male (*P* = 0.092) or in female (*P* = 0.512).

### Clinical characteristics of the study subjects

The clinical characteristics of the study subjects are shown in Table 1. Clinical, anthropometric, and metabolic parameters were analyzed according to the presence or absence of sarcopenia and obesity. The mean age of subjects was not significantly different in those without obesity regardless of their sarcopenic body fat classification, but those with obesity were significantly older when sarcopenia was accompanied (*P* = 0.0023). In addition, most of the continuous parameters except for HbA1c, T-chol, low-density lipoprotein cholesterol (LDL-C) and non-HDL-C, demonstrated statistically significant differences according to the presence or absence of sarcopenia. In case of HDL-C and serum creatinine, only those without obesity showed significant differences between those with or without sasrcopenia. The prevalence of DM, HTN and hyperlipidemia were significantly higher only in sarcopenia subjects without obesity, while the presence of sarcopenia did not affect their prevalences in obese subjects.

**Table 1:** Clinical characteristics of the study subjects according to the presence of sarcopenia and obesity.

	obesity (−)			obesity (+)		
	sarcopenia (−) (n = 900, Pop = 2,705,595)	Sarcopenia (+) (n = 176, Pop = 554,083)	*P*	Sarcopenia (−) (n = 224, Pop = 731,428)	Sarcopenia (+) (n = 278, Pop = 897,397)	*P*
age (years)	73.0±0.3	73.5±0.5	0.3597	71.4±0.4	72.9±0.4	0.0023
male (%)	44.8±1.6	59.1±4.4	0.0037	27.1±3.5	31.6±3.0	0.3497
ASM (kg)	16.6±0.1	15.6±0.3	0.0017	18.3±0.3	16.7±0.3	0.0002
ASM/weight (%)	30.8±0.2	27.1±2.3	<0.001	28.0±0.3	24.8±0.2	<0.001
BMI (kg/m^2^)	21.8±0.1	22.9±0.2	<0.001	26.7±0.1	27.6±0.1	<0.001
waist circumference (cm)	78.4±0.3	83.6±0.6	<0.001	90.3±0.4	93.7±0.6	<0.001
FPG (mg/dl)	100.9±0.9	116.7±4.2	<0.001	104.5±1.3	111.9±2.0	0.002
HbA1c (%)	7.1±0.1	7.4±0.2	0.146	7.0±0.1	7.2±0.2	0.176
HOMA2-IR	1.2±0.003	1.4±0.05	0.001	1.5±0.05	1.9±0.06	<0.001
T-chol (mg/dl)	188.5±1.5	192.2±3.8	0.374	191.3±2.4	198.0±2.7	0.058
TG (mg/dl)	133.0±3.8	156.2±5.4	<0.001	153.2±6.8	174.1±7.1	0.034
HDL-C (mg/dl)	46.7±0.5	44.2±0.9	0.006	42.7±0.6	43.6±0.7	0.376
LDL-C (mg/dl)	108.7±3.0	114.4±8.2	0.514	117.5±5.5	112.8±6.7	0.549
non-HDL-C (mg/dl)	141.8±1.4	147.9±3.8	0.133	148.6±2.3	154.4±2.5	0.084
serum creatinine (mg/dl)	0.8±0.01	0.9±0.01	0.014	0.9±0.01	0.9±0.01	0.407
smoking (%)	17.4±1.5	11.6±2.5	0.046	9.5±2.4	7.8±1.6	0.561
DM (%)	12.1±1.3	26.5±3.7	<0.001	22.6±3.2	24.9±2.8	0.592
HTN (%)	53.0±2.1	64.3±4.4	0.017	70.9±3.5	73.2±3.1	0.615
hyperlipidemia (%)	24.9±1.8	39.9±4.3	0.001	35.5±3.5	39.0±3.4	0.471
patients taking anti- diabetic medication (%)	10.7±1.3	25.1±3.8	<0.001	19.2±3.1	24.2±2.8	0.242
patients taking anti-hypertensive medication (%)	53.0±2.1	64.3±4.4	0.017	70.9±3.5	73.2±3.1	0.614
patients taking lipid-lowering medication (%)	4.5±0.7	9.4±2.6	0.075	9.4±2.3	8.7±2.0	0.817
CVD (%)	7.0±1.0	13.1±3.1	0.068	10.0±2.2	12.3±2.1	0.405
stroke (%)	3.9±0.8	6.6±1.8	0.183	5.6±1.7	6.2±1.5	0.825
CAD (%)	3.3±0.6	6.5±2.1	0.155	4.8±1.5	6.7±1.5	0.476

Data are mean ± standard error, or frequency (%).

*P*-values were analyzed using the Student's t-test between those with and without sarcopenia in non-obese and obese subjects, respectively.

Sarcopenia was defined as values less than 1SD below the sex-specific mean for ASM/weight in a healthy, younger person (20–39 years old).

Obesity was defined as BMI greater than 25 kg/m^2^.

Abbreviations: ASM, appendicular skeletal muscle mass; BMI, body mass index; FPG, fasting plasma glucose; HbA1c, hemoglobin A1c; HOMA2-IR, homeostasis model assessment 2 – insulin resistance; T-chol, total cholesterol; TG, triglyceride; HDL-C, high-density lipoprotein-cholesterol; LDL-C, low-density lipoprotein-cholesterol; DM, diabetes mellitus; HTN, hypertension; CVD, cardiovascular diseases; CAD, coronary artery disease.

### Association between sarcopenia and CVD

Spearman correlation analysis showed that ASM/weight correlated negatively with age (*r* = −0.112), waist circumference (*r* = −0.324), BMI (*r* = −0.497) and total cholesterol (*r* = −0.233, all *P*<0.001). ASM/weight correlated positively with HDL-C (*r* = 0.062, P = 0.039) (Table 2). Compared to non-sarcopenic subjects without MetS (M(−)S(−)), sarcopenic subjects with MetS (M(+)S(+)) had a higher prevalence of CVD (*P* = 0.008) (Table S1). The prevalence of CVD among those with MetS was higher when sarcopenia was also present with borderline significance (*P* = 0.059). In addition, those without MetS were shown to have a higher likelihood of concomitant CVD when they were sarcopenic.

**Table 2:** Multiple logistic regression analysis and backward selection for CVD.

Variables	OR (95% CI)	*P*
Hyperlipidemia with medication	3.026 (1.509–6.067)	0.002
Sarcopenia	1.768 (1.075–2.909)	0.025

Abbreviation: CVD, cardiovascular diseases.

Age, gender, diabetes, hypertension, hyperlipidemia, current smoking, obesity, serum creatinine, DM with medications, HTN with medication, hyperlidemia with medication and sarcopenia were included as dependent variables.

To determine the independent association between sarcopenia and CVD, multiple logistic regression analysis was performed (Table 2). The analysis included age, gender, DM, HTN, hyperlipidemia, current smoking, obesity, serum creatinine, DM with medications, HTN with medications, and hyperlipidemia with medications and sarcopenia as dependent variables. The results indicated that the odds ratio for CVD in those taking lipid lowering medication was 3.026 (95% CI 1.509–6.607) and 1.768 (95% CI 1.075–2.909) in those with sarcopenia (Table 2). However, obesity was found not to have a significant independent association with CVD (Data not shown).

## Discussion

In the present study, we found that the prevalence of sarcopenia among those older than 65 years according to KNHANES IV was 30.3% in men and 29.3% in women. There has been no consensus about the definition of sarcopenia. Baumgartner et al. defined sarcopenia as a ratio of ASM-to-body height in meters squared (ASM/height^2^) less than two standard deviations below reference values from young, healthy individuals measured with DXA [5] and reported that the prevalence was more than 50% in persons over 80 years of age. Davison et al. defined sarcopenia as muscle mass which was indirectly calculated using the bioelectrical impedance analysis equation, and reported that 1.7% of men and 2.8% of women in the older US population from NHANES III had sarcopenia [16]. Our study used the definition of sarcopenia where ASM was taken as a percentage of body weight (ASM/weight), which was modified from the studies of Janssen et al. [4] because sarcopenia defined by ASM/weight exhibited a more close association with metabolic parameters than when defined by ASM/height^2^ [23]. A previous study which used the same criteria for sarcopenia in an older Korean population reported that the prevalence of sarcopenic obesity was 35.1% in men and 48.1% in women [23]. It is speculated that the difference in prevalence of sarcopenia is mainly due to the lack of consensus of its definition. It was interesting to notice that, unlike previous findings [28], [29], the prevalence of sarcopenia appeared to decrease with increasing age older than 80 years as shown in Figure 1. It is speculated that the small number of participants older than 80 years may have contributed to this discrepancy. Though not significant, the p trend for prevalence of sarcopenia showed some increasing trend in male (p = 0.092), but female did not show such significance. This may be due to a small number of female subjects especially at the age between 80 and 84. A large scaled study may resolve this issue.

**Figure 2:**
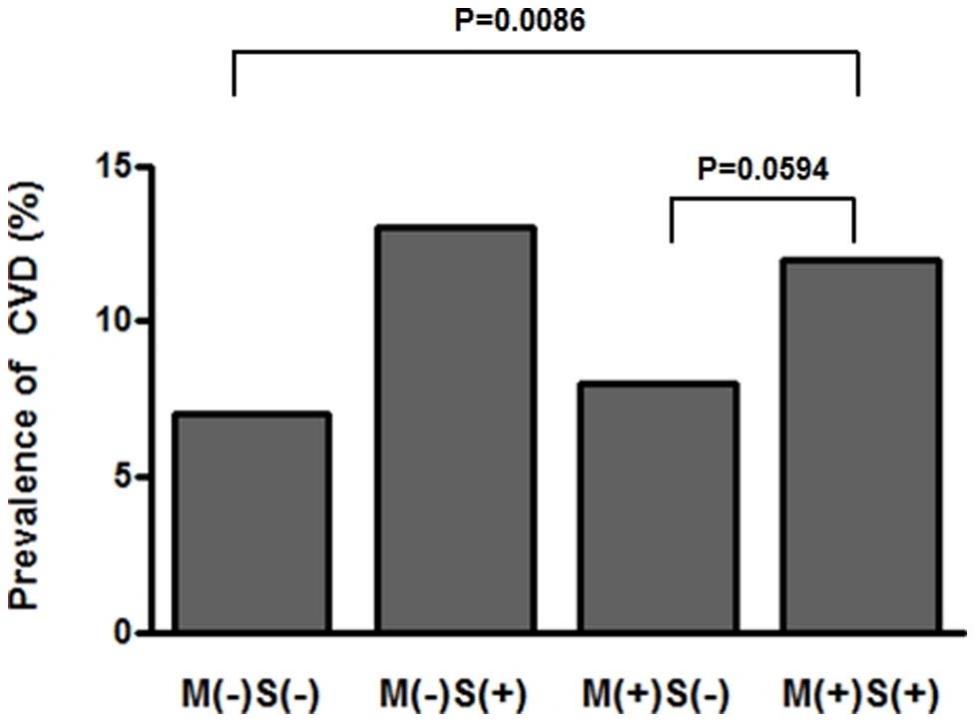
CVD prevalence according to the presence of metabolic syndrome and sarcopenia. Abbreviation: CVD, cardiovascular diseases; M, metabolic syndrome; S, sarcopenia.

Our study showed that sarcopenia was associated with cardiovascular events independent of other well-known CVD risk factors, which is in agreement with previous studies reporting the independent association between sarcopenia and CVD. Sanada et al. analyzed the data from the general Japanese population and reported that sarcopenia was associated with greater arterial stiffness in women, implying that sarcopenia is associated with risk factors for CVD [14]. Low 24 h urinary creatinine excretion rate, which has been shown to indicate the presence of sarcopenia, was associated with major adverse cardiovascular events and all-cause mortality in the general population [18], [19]. Our data also demonstrated that those with obesity and sarcopenia had a higher tendency toward CVD than those with obesity only (12.3±2.1% vs. 10.0±2.2%, Table 1). In addition, those with MetS were more likely to have CVD when sarcopenia was also present, and surprisingly those without MetS were also more likely to experience CVD when they had sarcopenia (Figure 2). These findings suggest that sarcopenia may be an independent risk factor for CVD. On the contrary, obese and sarcopenic postmenopausal women have been reported to have a lower risk of CVD than obese postmenopausal women without sarcopenia, possibly implying a protective effect of sarcopenia [15]. Sarcopenia in combination with obesity was not found to place older individuals at higher risk for functional limitations [16]. However, it should be mentioned that these results were derived from specific groups such as postmenopausal women [15], and skeletal muscle mass was estimated with predictive equations (skeletal muscle (kg)  =  [(Ht2/*R* ×0.401) + (sex ×3.825) + (Age in years ×−0.071)] +5.102, where Ht  =  height in cm, *R*  = resistance in ohms from bioelectrical impedence analysis, and sex  = 0 for women and 1 for men) [16] instead of direct measurement methods such as DXA.

It has been suggested that sarcopenia is not an isolated event but is accompanied by a simultaneous increase in fat mass [30]. This lipid infiltration plays a role in sustaining sarcopenia through a macrophage mediated-release of pro-inflammatory cytokines and adipokines from adipocytes which induce chronic inflammation [31]. Those with sarcopenia also commonly experience functional impairment and physical disability [4], [32] which causes a reduction in muscle contraction-induced factors having an anti-inflammatory effect, the so-called myokines [33]. The relative paucity of myokines in sarcopenia may increase the risk of CVD [34]. Altogether, functional deterioration and chronic inflammation as well as the reduction of anti-inflammatory substances observed in those with sarcopenia contribute to the development of insulin resistance, type 2 DM, hyperlipidemia and HTN [13], and eventually raises the CVD risk.

On the other hand, it should be also noted that our analyses did not demonstrate statistically significant correlation between ASM/weight and HOMA2-IR (Table S1). In addition, the multiple logistic regression analysis exhibited a significant association between sarcopenia and CVD even after adjustment for obesity (Table 2). Altogether, it is plausible that obesity in older populations may not be a major factor which explains most of the association between sarcopenia and CVD. This is conflicting to a previous study suggesting that obesity-associated inflammation leads to sarcopenia in older populations [35]. Additional studies with a larger number of subjects and different population groups should assess possible mechanisms other than insulin resistance which could explain the association between sarcopenia and CVD.

The major limitation of this study was the cross-sectional design that precluded addressing the issue of causation. In addition, since we did not perform any imaging work-up such as coronary angiogram or brain MRI to verify the presence of stroke or coronary heart disease, there might be a number of undiagnosed subjects with CVD, leading to an underestimate of the prevalence of CVD. Despite these limitations, the major strength of this study is that our findings were based on the data from a general population study in Korea including ASM which was directly measured with DXA. In addition, most of the previous studies focused on the role of sarcopenic obesity as another risk factor for CVD but not sarcopenia itself. To our knowledge, our study is the first dataset available emphasizing the significant association of sarcopenia alone with CVD among the general population older than 65 years in Korea.

In conclusion, this study highlighted that sarcopenia was associated with CVD independent of other well-documented cardiovascular risk factors, renal function and medications in elderly Korean adults. Efforts must be made to prevent and treat sarcopenia in the older population, which would also decrease the risk of CVD.

## Supporting Information

Table S1 **Spearman correlation analysis with ASM/weight.**
(DOC)
