# Supplementary material for: Sarcopenia Is Independently Associated with Cardiovascular Disease in Older Korean Adults: The Korea National Health and Nutrition Examination Survey (KNHANES) from 2009
Source: PLoS One. 2013 Mar 22;8(3):e60119. doi: 10.1371/journal.pone.0060119 (PMC3606314; doi:10.1371/journal.pone.0060119)
Supplement: Table S1 — Spearman correlation analysis with ASM/weight. (DOC) [file pone.0060119.s001.doc]

**Table S1** Spearman correlation analysis with ASM/weight

| **Variables** | ***r*** | ***P*** |
| --- | --- | --- |
| Age | -0.112 | 0.001 |
| Waist circumference | -0.324 | <0.001 |
| BMI | -0.497 | <0.001 |
| FPG | -0.1667 | 0.043 |
| HOMA-2 | -0.3902 | 0.508 |
| T-chol | -0.233 | <0.001 |
| TG | -0.2302 | 0.379 |
| HDL-C | 0.062 | 0.039 |
| LDL-C | -0.152 | 0.05 |

Abbreviations: ASM, appendicular skeletal muscle mass; BMI, body mass index; FPG, fasting plasma glucose; HOMA-2, homeostasis model assessment -2; T-chol, total cholesterol; TG, triglyceride; HDL-C, high-density lipoprotein-cholesterol; LDL-C, low-density lipoprotein-cholesterol
